# A Comprehensive Study of Object Tracking in Low-Light Environments

**DOI:** 10.3390/s24134359

**Published:** 2024-07-05

**Authors:** Anqi Yi, Nantheera Anantrasirichai

**Affiliations:** Visual Information Laboratory, Bristol BS1 5DD, UK; lm20690@bristol.ac.uk

**Keywords:** tracking, low-light enhancement, denoising

## Abstract

Accurate object tracking in low-light environments is crucial, particularly in surveillance, ethology applications, and biometric recognition systems. However, achieving this is significantly challenging due to the poor quality of captured sequences. Factors such as noise, color imbalance, and low contrast contribute to these challenges. This paper presents a comprehensive study examining the impact of these distortions on automatic object trackers. Additionally, we propose a solution to enhance the tracking performance by integrating denoising and low-light enhancement methods into the transformer-based object tracking system. Experimental results show that the proposed tracker, trained with low-light synthetic datasets, outperforms both the vanilla MixFormer and Siam R-CNN.

## 1. Introduction

The task of visual-based object tracking has been a core research area in computer vision for decades, focusing on determining the state of a designated target within video sequences, starting from its initial state. Its applications include surveillance, biometric recognition, security, robotics, automotive, transportation, ethology, etc. However, tracking objects in low-light environments presents significant challenges due to the poor sequence quality captured. The presence of noise, motion blur, color imbalance, and low contrast in these sequences makes it difficult for traditional algorithms to accurately track objects. This paper explores methods aimed at enhancing the performance of visual object tracking in low-light conditions and analyzing how various factors, such as noise, color imbalance, and low contrast, impact tracking effectiveness.

Similar to other computer vision tasks, deep learning has emerged as an effective tool for object tracking. Early learning-based methods adapted object recognition techniques to individual frames within a video [1]. Subsequently, recurrent neural networks (RNNs) were integrated to track detected objects over time. In 2017, the transformer was introduced [2], proposing a novel architecture for natural language processing tasks that solely relies on attention mechanisms, eliminating the need for recurrent or convolutional neural networks (CNNs). The key innovation of the transformer architecture is its ability to process input sequences in parallel, rather than sequentially as in traditional RNN models. This property enables more efficient training and faster convergence. Additionally, the transformer model effectively handles long-range dependencies in input sequences, addressing a common issue faced by RNNs, which has made it an attractive option for object tracking.

This paper employs a state-of-the-art approach for transformer-based object tracking, MixFormer [3], known for its superiority over several CNNs, RNNs, and earlier transformer models. MixFormer simplifies the traditional multi-stage pipeline and integrates feature extraction and target information integration within a unified transformer-based framework. In contrast to existing trackers, such as the Siam R-CNN [4], which relies on CNNs pretrained for generic object recognition, MixFormer leverages the flexibility and global modeling capacity of attention operations to capture target-specific features and promote wide range communication between the target and search area. By introducing a Mixed Attention Module (MAM) with hybrid interaction schemes, MixFormer enables simultaneous feature extraction and target integration, which results in a more compact and neat tracking pipeline. This approach overcomes the limitations of traditional trackers that use separate components for feature extraction, integration, and target-aware localization. The experiments in [3] demonstrate that MixFormer outperforms 38 existing methods tested on four benchmarking datasets. Recently proposed, DiffusionTrack [5] employs a diffusion model to approach object tracking as a generative task over the space of point sets within the search region. It reports a state-of-the-art performance; unfortunately, the code and pretrained model are not publicly available.

In low-light conditions, using the tracker faces several limitations. (i) Lack of specialized modules for low-light tracking: The Mixformer is specifically designed to capture target features in daylight conditions. However, these features may become indistinct or distorted in low-light conditions due to insufficient lighting and noise, thereby limiting its performance. (ii) Limited training data in low-light conditions: The model relies on a substantial amount of labeled data to effectively conduct visual object tracking. The inadequacy of training data specific to low-light conditions contributes to diminished performance in such scenarios. To address these issues, we propose integrating a denoiser and enhancement module to the framework and using synthetic low-light datasets to train the model.

This paper presents a comprehensive study on object tracking in low-light environments. We investigated the distinct types of distortions present in low-light content and their individual impacts on the tracking performance. Subsequently, we enhanced the trackers by employing preprocessing techniques involving denoising and brightness enhancement. Finally, we discuss the limitations of the current approach. More specifically, the contributions of this paper are listed as follows:

- We created an end-to-end framework applying denoising and enhancement to object tracking in low-light environments.
- The proposed framework was trained with synthetic data. Therefore, three loss functions can be used to constrain the three modules, i.e., denoiser, enhancer, and tracker, to sequentially learn to suppress noise and enhance features to an appropriate form for object tracking.
- We studied the influence of low-light distortions, including noise levels, gamma, and saturation gains, on the performance of our object tracker.
- We tested four object tracking methods, including MixFormer [3], Siam R-CNN [4], VideoTrack [6], and DETR [7], when integrated into our end-to-end framework.

## 2. Related Work

### 2.1. Object Tracking

The early work in learning-based object detection focused on fully convolutional networks (FCNs), which have demonstrated effectiveness in capturing both local and global contextual information during the tracking process [8]. Subsequently, more sophisticated methods gained popularity, such as the fully convolutional Siamese network [9]. Further enhancements include the Siamese Region Proposal Network (SiamRPN) [10], which integrates the Siamese network with a region proposal mechanism for high-performance tracking. Additionally, Discriminative Model Prediction (DiMP) [11] was proposed to address object deformations and occlusions during tracking. Recently, the work integrated with a non-local means block in [12] has shown a significant improvement for instant segmentation in low-light scenes.

The transformer architecture and attention mechanisms have recently emerged as powerful techniques in various computer vision tasks, including object tracking. The transformer architecture has successfully replaced traditional convolutional layers with self-attention mechanisms. A well-known example of a transformer-based object tracking model is DETR (DEtection TRansformer) [7], which captures both local and global context information, enabling it to handle complex scenarios effectively. Although DETR is primarily focused on object detection, it can be adapted for object tracking tasks by gathering information from multiple frames. Another example is TrackFormer [13] for multi-object tracking. This single unified transformer architecture performs both detection and tracking in an end-to-end manner. The model demonstrates exceptional performance in multi-object tracking benchmarks. Similarly, the MOTR model [14] employs a transformer-based architecture with a temporal aggregation network for multiple-object tracking.

In addition to transformer-based models, attention mechanisms have been integrated into other object tracking models to enhance their performances. This includes the Distractor-aware Siamese Networks (DaSiamRPN) [15], where a distractor-aware training strategy incorporates attention mechanisms to search objects effectively. This strategy improves the tracker’s robustness against distractors. The Attentional Correlation Filter Network (ACFN), proposed in [16], incorporates an attention mechanism into a correlation filter-based tracker to adaptively weigh different spatial regions based on their importance during tracking.

We also note here that there are attempts to apply object detection to each frame of the video [17,18]; however, this approach does not inherently track objects across frames. Consequently, it can sometimes miss tracking an object if the detection fails in some frames. The main advantage of these track-by-detection methods is their speed. A comprehensive review can be found in [19].

### 2.2. Low-Light Enhancement

The advancements in deep learning have significantly progressed image enhancement, yet learning-based video enhancement remains relatively new. Some methods show promise in extending low-light image enhancement techniques to videos. These strategies involve estimating noise maps to guide attention mechanisms, implementing self-calibrated illumination frameworks, and utilizing adaptive total variation regularization (e.g., [20,21]). Additionally, there is growing interest in techniques that integrate the Retinex theory model with learnable physical priors for reflectance and illumination (e.g., [22]). Diffusion models (DMs) have gained popularity for enhancing low-light images [23,24,25]. Diff-Retinex [24] leverages Retinex decomposition alongside multi-path generative diffusion networks to reconstruct the normal-light Retinex probability distribution. Another recent method [25] utilizes a wavelet transform to separate images into high and low frequencies; the high frequencies are enhanced via a transformer-based pipeline, while the low frequencies are processed through diffusion. Despite their excellent performance, DMs are notably slow to train and demand substantial memory.

For video processing, various methods utilize alignment modules (e.g., [26]) to synchronize the feature maps of neighboring frames with the current frame, aiding in motion handling. Despite their purpose, these alignment modules occasionally fall short in compensating for motion adequately, leading to artifacts in feature combinations. Some approaches leverage Siamese Networks with shared weights to reduce noise in videos [27]. STA-SUNet [28] has demonstrated that using transformers for low-light video enhancement outperforms CNN-based methods [29]. To cope with limited paired datasets, certain methods resort to unpaired training strategies, such as employing CycleGAN [30].

### 2.3. Object Detection in Low-Light Environments

Although no tracking methods are specifically proposed for low-light environments, some methods have been developed for detection, particularly using YOLO [31]. For instance, Retinex has been integrated with YOLO for urban surveillance [32]. Similarly, IDOD-YOLOV7 [33] combines the optimal learning of an image defogging module with YOLO for object detection in low-light, foggy traffic environments. Additionally, similar to our proposed framework, HighlightNet [34] introduces a preprocessing network for UAV trackers.

## 3. Methods for Object Tracking in Low-Light Environments

**Figure 1:**
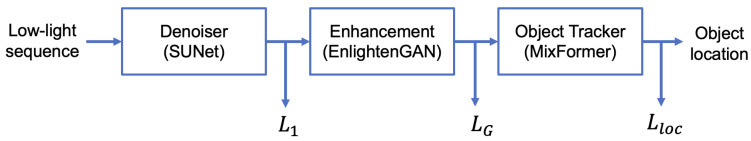
Diagram for our study on object tracking in low-light scene.

Despite the existence of specific methods proposed for low-light enhancement, we chose to adopt separate denoising and light correction approaches. This decision allows us to investigate the distinct impacts of various distortions on the tracker’s performance within low-light environments. The workflow, depicted in Figure 1, integrates the MixFormer tracker with two preprocessing modules. Depending on the specific cases studied, one or both of these preprocessors might be omitted. Moreover, with the utilization of synthetic low-light datasets, we have access to clean, daylight ground truth data, enabling us to fine-tune the networks.

### 3.1. Preprocessing with Denoising

In visual object tracking tasks, noise is inevitable and can significantly impact tracking efficiency. A common solution to address this issue is to preprocess tracking data before inputting them into the tracking network. Denoising techniques, such as filtering, temporal accumulation, and learning-based methods, are widely used in practice [35,36,37].

In this study, we adopted the state-of-the-art method SUNet [38] for denoising. This model, although simple, effectively combines the Swin Transformer and UNet architectures, enhancing feature extraction and hierarchical representation capabilities. Its dual up-sample block architecture, employing subpixel and bilinear up-sampling methods, helps prevent checkerboard artifacts and enhances overall performance. Demonstrating competitive results on widely-used denoising datasets, the SUNet model proves its practical effectiveness in addressing real-world image denoising issues, reporting better performances than CNN-based approaches (e.g., [39,40]) and results comparable with those if SwinIR [41], which is also a Swin Transformer-based approach, but SUNet is faster. In this project, a pretrained SUNet model was utilized to preprocess the input dataset via denoising, aiming for an improved tracking performance.

### 3.2. Preprocessing with Enhancement

In the previous sections, a methodology was discussed for addressing noise in low-light sequences. However, other low-light features, such as color imbalance and low contrast, also contribute to the degradation of the tracking performance. Various light-enhancement methods have been proposed in the past, ranging from histogram based ones to learning based ones.

Here, we adopted EnlightenGAN [42], which is a deep learning-based generative adversarial network. The model represents a significant advancement in the field, introducing a pioneering unpaired training strategy that eliminates the need for paired training data and improves real-world generalization. Its innovative global-local discriminator structure addresses spatially varying light conditions effectively, while self-regularization techniques, including self-feature preserving loss and self-regularized attention mechanisms, contribute to the model’s success in the unpaired setting. EnlightenGAN offers superior performance and adaptability in comparison to state-of-the-art methods. From the diagram in Figure 1, when the EnlightenGAN is fine-tuned, the least-square GAN loss $L_{G}$ is applied to the generator of the EnlightenGAN.

### 3.3. MixFormer

MixFormer [3] tracks the target object by progressively extracting coupled features for the target template and search area while deeply integrating the information between them. This architecture consists of two main components: (i) a backbone, which comprises iterative target-search MAMs (mixed attention mechanisms), and (ii) a localization head, which is responsible for producing the target bounding box. The MAM blocks allow for the simultaneous extraction and integration of features from the target template and search area. The localization head simplifies the process of localizing the tracked object within the search area, making the overall pipeline more efficient.

One of the key advantages of the MixFormer model is its compact and neat tracking pipeline. Unlike other trackers that typically decouple the steps of feature extraction and information integration, MixFormer combines these steps within its single backbone. This design choice results in a more efficient and streamlined architecture. Additionally, the MixFormer model does not require an explicit integration module or any post-processing steps, further simplifying the overall tracking pipeline. This simplification can lead to reduced computational complexity and faster inference times, making the MixFormer model a more suitable option for real-time tracking applications.

**The Mixed Attention Module** (MAM) processes the input target template and search area with the aim of simultaneously extracting their long-range features and fusing the information interaction between them. This module enhances the tracker’s ability to capture and integrate essential information from both the target and search area smoothly. Unlike the original multi-head attention mechanism [2], the MAM operates on two separate token sequences corresponding to the target template and search area. It achieves this through dual attention operations. Self-attention is performed on the tokens (image patches) in each sequence (target and search) themselves to capture target- or search-specific information. Cross-attention is conducted between tokens from two sequences to allow communication between the target template and the search area. A concatenated token sequence is used to implement the mixed attention mechanism. Let vectors $q_{t}$, $k_{t}$, and $v_{t}$ represent targets $q_{s}$, $k_{s}$, and $v_{s}$ to represent the search region. The mixed attention can be defined as follows:

(1) $$k_{m}=Concat\left(k_{t},k_{s}\right),\;v_{m}=Concat\left(v_{t},v_{s}\right),$$

(2) $$Attention_{t}=softmax\left(\frac{q_{t}k_{m}^{T}}{\sqrt{d}}\right)v_{m},$$

(3) $$Attention_{s}=softmax\left(\frac{q_{s}k_{m}^{T}}{\sqrt{d}}\right)v_{m},$$

where *d* denotes the dimension of the key vectors, and $Attention_{t}$ and $Attention_{s}$ are the attention maps of the target and search, respectively.

To achieve the additional modeling of local spatial context, a separable depth-wise convolutional projection layer is performed on each feature map (i.e., the query, key, and value). Then, each feature map of the target and search is flattened and processed by a linear projection to produce queries, keys, and values of the attention operation. Finally, the target token and search token are concatenated and processed by a linear projection.

**The online template update** plays a crucial role in capturing temporal information, as well as addressing object deformation and appearance variations in visual tracking. However, it is widely acknowledged that poor-quality templates may result in a inferior tracking performance. Consequently, the authors introduced a score prediction module (SPM) to select reliable online templates based on their predicted confidence scores.

The SPM comprises two attention blocks and a three-layer perceptron. Initially, a learnable score token serves as a query to attend to the search ROI (region of interest) tokens. This process enables the score token to encode the extracted target information. Subsequently, the score token attends to all positions of the initial target token, implicitly comparing the extracted target with the first target. Finally, the score is generated by the MLP (multi-layer perceptron) layer and a sigmoid activation function.

The online template is considered negative when its predicted score falls below 0.5. By filtering out low-confidence templates, the SPM helps improve the overall tracking performance. The introduction of the SPM ensures that the tracker utilizes high-quality templates for tracking, which in turn enhances its ability to adapt to object deformation and appearance changes. This approach enables a more accurate and robust tracking performance in various challenging scenarios.

**The loss function** used by the MixFormer model is a combination of L1 loss and GIoU loss. It is denoted as follows:

(4) $$L_{loc}=\lambda_{L1}L1\left(B_{i},\hat{B}\right)+\lambda_{GIoU}L_{GIoU}\left(B_{i},\hat{B}\right)$$

where $\lambda_{L1}=5$ and $\lambda_{GIoU}=2$ are the weights of the two losses, $B_{i}$ is the ground-truth bounding box, and $\hat{B}$ is the predicted bounding box. L1 loss is commonly used because of its robustness and insensitivity to outliers. Object tracking often involves dealing with occlusions, sudden motion changes, and noisy measurements. L1 loss is less sensitive to outliers because it considers the absolute differences; thus, it is more robust and is an ideal choice for visual object tracking tasks.

Generalized Intersection over Union (GIoU) loss $L_{GIoU}$ was designed to address the limitations of the commonly used Intersection over Union (IoU) metric as it does not provide meaningful gradients for non-overlapping bounding boxes [43]. GIoU loss addresses this issue by extending the IoU metric to account for the non-overlapping bounding boxes as well. It is computed as follows:

(5) $$GIoU=IoU-\frac{\left|C\setminus (A\cup B)\right|}{\left|C\right|}=\frac{\left|A\cap B\right|}{\left|A\cup B\right|}-\frac{\left|C\setminus (A\cup B)\right|}{\left|C\right|}$$

where *A* and *B* are the prediction and ground-truth bounding boxes, and *C* represents the area of the smallest enclosing box containing both boxes.

For the online training stage, a standard cross-entropy loss is used to train the SPM. It is defined as follows:

(6) $$L_{score}=y_{i}\log\left(p_{i}\right)+\left(1-y_{i}\right)\log\left(1-p_{i}\right)$$

where $y_{i}$ is the ground-truth label, and $p_{i}$ is the predicted confidence score.

## 4. Experiments and Discussion

### 4.1. Synthetic Low-Light Dataset

We used the GOT-10K dataset [44], which is a large-scale, high-diversity benchmark for visual object tracking, comprising a wide variety of sources, such as YouTube, Vimeo, and Dailymotion. GOT10K contains more than 10,000 videos and covers 560 distinct object classes. The predefined testing set consists of 420 videos, including 84 different object classes and 31 forms of motion. To prevent larger-scale classes from dominating the evaluation results, the maximum number of videos for each class was limited to 8, which accounts for only 1.9% of the test set size. The validation set was created by randomly sampling 180 videos from the training subset, with a uniform probability distribution across different object classes.

The GOT-10K dataset was captured in normal light and good conditions, whereas video sequences taken in poor lighting conditions often display attributes like low brightness and contrast, a limited grayscale spectrum, color distortion, and considerable noise. To synthesize low light, we followed the image degradation model proposed in [45], and we included the color imbalance effect C in the model, as shown in Equation (7):

(7) $$g\left(x,y\right)=C\left(\alpha \cdot f{\left(x,y\right)}^{\gamma }+\beta \right)+\epsilon_{n},$$

where g(x,y) is the output image, f(x,y) is the input image, α is the contrast adjustment parameter, β is the brightness adjustment parameter, γ is the gamma factor, and $\epsilon_{n}$ represents Gaussian noise.

An α value above 1 boosts image contrast, darkening dark areas and brightening bright areas. An α value below 1 reduces image contrast, lightening dark areas and darkening bright areas. An α value of 1 maintains the image’s contrast unchanged. A positive β increases image brightness, a negative β decreases it, and β at 0 maintains the brightness. The γ value describes the nonlinearity of the imaging system to different input brightness levels. Typically ranging from 0.1 to 5, a gamma of 1 signifies a linear relationship between input and output brightness. A gamma above 1 accentuates sensitivity to darker areas, while that below 1 emphasizes brighter areas.

Creating a color imbalance effect C in dark images can be accomplished by selectively manipulating the saturation channel (*S*) without altering the hue (*H*) or value (*V*) channels. This is achieved by applying a scaling factor to the saturation channel, which can be represented by the equation $S^{\prime }=S\cdot \alpha_{S}$, where $S^{\prime }$ is the adjusted saturation, *S* is the original saturation, and $\alpha_{S}$ is the scaling factor. In selectively adjusting the saturation of specific color channels, an imbalance in the color distribution can be created that mimics the appearance of the color imbalance often observed in real-world low-light conditions. Note that modifying the *V* channel alone will not achieve color imbalance, as it only affects the overall brightness of the image without altering the color relationships. We refrained from adjusting *H* as it tended to alter the white balance, which is a task already effectively addressed by commercial software.

Finally, we added Gaussian noise with the mean μ = 0 and the standard deviation σ, determining the spread or the variability of the noise added to the image. A larger standard deviation implies that the image is more “grainy” or “fuzzy” due to the presence of more random noise values.

### 4.2. Training Setting

We trained the models with various synthetic low-light data with diverse parameters, including Gaussian noise (σ) and gamma (γ) adjustment and saturation adjustment scaling factor ($\alpha_{S}$). These trackers, along with the tracker attained by normal light, were tested on a single synthesized dark test set to evaluate the tracking results of training with different parameters and to assess the impact of different low-light features on the tracking accuracy of the tracker. The range of the parameters were set as below. These ranges were determined through observation during testing with a tracker trained on a normal-light dataset, covering a wide range of tracker performances, including the following:

- For Gaussian noise, the mean value was constant at 128 for all datasets, while the σ was set to 10, 25, 40, 55, and 70, with the default being 10.
- For contrast adjustment, the linear intensity factor was maintained at 0.4 for all datasets, while the γ was set to 0.2, 0.3, 0.4, 0.5, and 0.6, with the default being 0.5.
- For saturation adjustment, the scaling factor $\alpha_{S}$ was set to 0.2, 0.3, 0.4, 0.5, and 0.6, with the default being 0.4.

It is worth noting that to assess the impact of each parameter on the tracking results, only one parameter was altered at each time, with the others set to their default values. Other training parameters remained the same as they were set for normal light to avoid the results being affected by other factors.

### 4.3. Metrics

**The Intersection over Union (IoU)** is calculated as the ratio of the intersection of the predicted and ground-truth regions to their union. In other words, it measures the overlap between the two regions, where a value of 1 indicates a perfect match and a value of 0 indicates no overlap.

**The Area Under the Curve (AUC)** refers to the area under the curve, plotting the fraction of successfully predicted frames against a threshold of IoU values. A higher AUC value suggests a better tracking performance as it indicates that the tracker is able to successfully track objects with a larger IoU threshold *t*, which ranges from 0 to 1. The AUC can be calculated using numerical integration and defined as follows:

(8) $$AUC=\int_{0}^{1}\frac{Number\;of\;frames\;with\;IoU>=t}{Total\;number\;of\;frames}\;dt$$

**OP50 and OP75** are the Overlap Percentages when thresholds (as percentages) of 50 and 75 are considered successful, respectively. Typically, OP75 is considered a more strict criterion for measuring the performance as it sets a higher threshold for the overlap percentage. A higher OP50 or OP75 implies a better performance.

**Precision** measures the accuracy of the predicted position of the tracked object. It calculates the average distance between the center of the ground-truth bounding box and the center of the predicted bounding box for each sequence [46]. The precision is the proportion of frames of which the distance is below a threshold *d*:

(9) $$precision\left(d\right)=\frac{Number\;of\;frames\;with\;distance_{i}<=d}{Total\;number\;of\;frames}$$

Here, $distance_{i}$ is the Euclidean distance between the center points in frame *i*:

(10) $$distance_{i}=\sqrt{{\left(x_{gt_{i}}-x_{pred_{i}}\right)}^{2}+{\left(y_{gt_{i}}-y_{pred_{i}}\right)}^{2}}$$

where $x_{gt_{i}}$ and $y_{gt_{i}}$ are the coordinates of the center of the ground-truth bounding box, and $x_{pred_{i}}$ and $y_{pred_{i}}$ are the coordinates of the center of the predicted bounding box.

**Normalized precision** takes into account the differences in object sizes and frame resolution by normalizing the distance between the ground truth and predicted bounding box centers. The normalization is commonly performed by dividing the $distance_{i}$ by the diagonal length of the ground-truth bounding box, which can be defined as follows [46], where *d* is the threshold:

(11) $$Normalized\;Distance_{i}=\frac{distance_{i}}{Diagonal\;length\;of\;ground\;truth\;bounding\;box_{i}}$$

(12) $$Normalized\;Precision\left(d\right)=\frac{Number\;of\;frames\;with\;Normalized\;distance_{i}<=d}{Total\;number\;of\;frames}$$

### 4.4. Impact of Low-Light Distortions on Tracking Performance

This section investigates the impact of individual distortions observed in low-light environments—such as noise, gamma, and saturation changes—on tracking performance. This demonstrates the parameters that should be set to generate synthetic low-light videos for training the model to achieve optimal performance when used in a general scenario.

#### 4.4.1. Noise Levels

**Figure 2:**
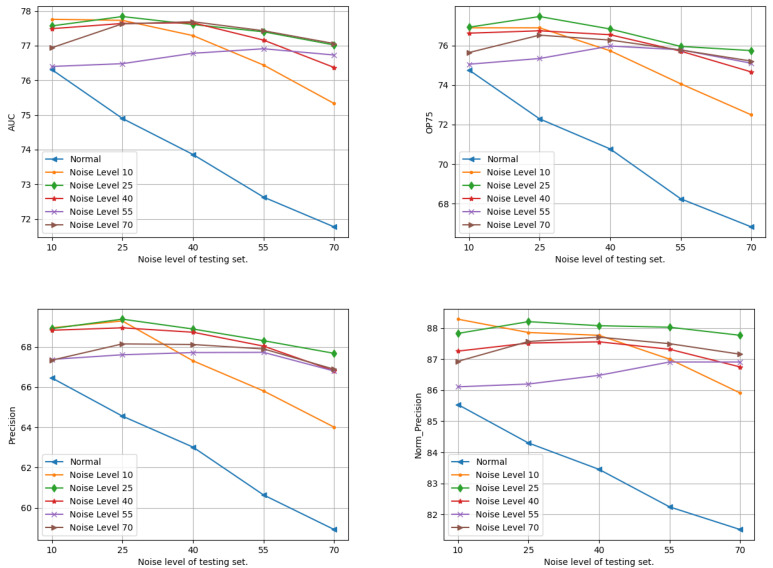
Test results of trackers trained with different noise levels. The x axis shows the noise level of the test sets, while the y axis shows the values of the testing metrics.

We explored the impact of varying noise levels on the tracker’s performance. While maintaining normal lighting conditions, we adjusted noise levels by generating test sets with different sigma values: 10, 25, 40, 55, and 70 for each set. All other parameters were maintained at their default values as specified in Section 4.2. The results are shown in Figure 2. When the model was trained in normal light without noise, it showed poor robustness to noise (as indicated by the blue line in the plots). Surprisingly, the model trained with a noise level of 25 demonstrates the highest performance in object tracking across varying noise levels. Conversely, models trained with higher noise levels failed to achieve an optimal tracking performance, even when tested under similar noise conditions. This struggle may indicate that the network faces difficulties in capturing features from very noisy inputs.

#### 4.4.2. Gamma Values

**Figure 3:**
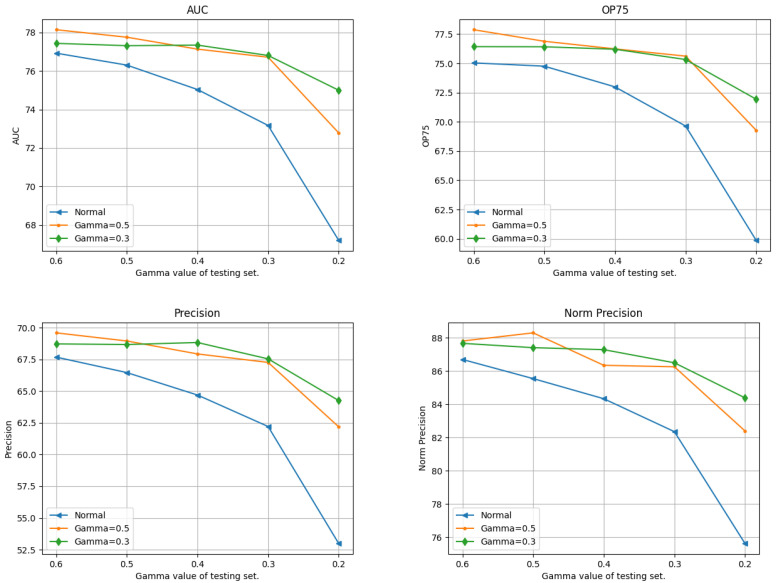
Test results of trackers trained with different gamma gains. The x axis shows the gamma value of the test sets, while the y axis shows the values of the testing metrics.

**Figure 4:**
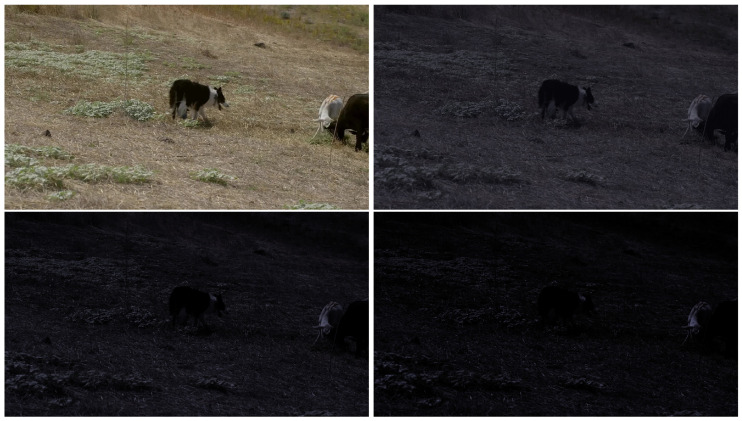
Images with different levels of gamma gain. Top left shows image in original daylight environment. Other images have gamma gains of 0.6, 0.3, and 0.2, respectively.

Figure 3 illustrates the test results of the three trackers trained with different gamma values. A notable observation is that the testing result of the model trained with daylight dataset exhibits a non-linear decrease. Specifically, in between gamma gains of 0.2 and 0.3 the model’s precision experienced a sharp decline. The same trend can be found in trackers trained with gamma gains of 0.3 and 0.5. This phenomenon can be explained by the characteristics of gamma correction. When the gamma gain decreases to a certain level, the image becomes extremely dark; hence, the object is not visually distinguishable, and it is also difficult for machines to extract useful features. Unlike noise, which distorts edges and destroys certain features, low brightness and low contrast cause the object to blend into the background, making it impossible to identify edges or features. Figure 4 shows the original daylight image in comparison to the synthesized outcome of images with gamma gains of 0.6, 0.3, and 0.2.

#### 4.4.3. Saturation Values

**Figure 5:**
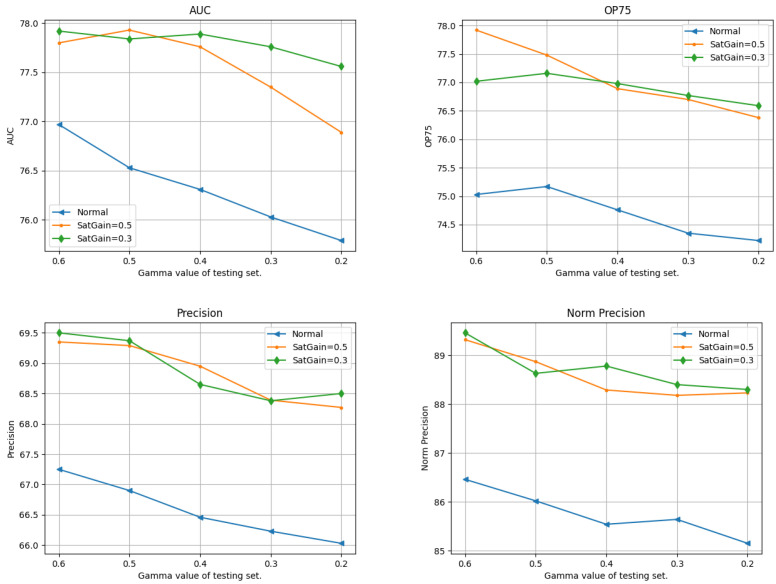
Test results of trackers trained with different saturation gains. The x axis shows the saturation gain values of the test sets, while the y axis shows the values of the testing metrics.

As shown in Figure 5, a descending pattern can be found in the curves as the saturation gain reduces. Furthermore, the trackers trained with saturation gains of 0.3 and 0.5 display a significantly improved performance compared to the tracker trained on the daylight dataset. This observation aligns with the previously mentioned findings regarding the impact of noise and gamma gain, where trackers trained on the synthetic low-light dataset show better robustness when tested on various dark datasets. This consistency suggests that training the model on datasets with diverse low-light features can improve their versatility and effectiveness when handling visual object tracking tasks in a wide range of low-light scenarios.

The impact of saturation on the model’s tracking efficiency is relatively smaller than that of noise and gamma gain. As the saturation gain drops from 0 to 0.2, these five metrics only decrease by approximately 3%. For the impact of noise, the AUC decreases from 79.30% to 73.86% as the noise level rises from 0 to 40. Similarly, OP50, OP75, precision, and normalized precision decline by 5.69%, 9.55%, 9.37%, and 5.48%, respectively. Similarly, when the gamma value drops from 1 to 0.3, while the features remain recognizable, the AUC, OP50, OP75, precision, and normalized precision decrease by 6.13%, 6.8%, 10.68%, 10.08%, and 6.58%, respectively.

While changes in saturation can alter the appearance of an image by adjusting the color intensity, they do not have massive impact on the overall image quality, or the visibility of the objects and their edges. This implies that alterations in shapes and edges deteriorate the tracking performance more significantly than saturation changes do. Thus, the model is able to maintain a high performance and is less affected by the changes in saturation compared to noise and gamma, where the features in the object are greatly impacted.

### 4.5. Ablation Studies

Table 1 shows the test results of trackers trained using synthetic dark datasets (sigma = 40, gamma = 0.5, saturation = 0.4). In using some modules of the proposed pipeline, the outcomes when only the denoiser was used surpass those when only the enhancement module was used, confirming that preprocessing with denoising significantly enhances the model’s performance compared to enlightening. Moreover, upon integrating the denoiser, the AUC increased by 5.36% compared to the original MixFormer. Additionally, we found that applying preprocessing solely to the testing set resulted in only a 3.21% increase in the AUC. This suggests that applying preprocessing to both training and testing procedures yields a more substantial improvement in the model’s performance.

### 4.6. Visualized Tracking Results

In this section, the visualized tracking results are discussed to further investigate the model’s ability in handling challenging conditions. Specifically, the reasons to why the tracking failed in certain cases are examined to provide insights into the future improvements of the model. The main reasons for the model’s failure to track objects are classified into three categories: (i) ambiguity caused by the background, (ii) presence of multiple, visually similar objects within the scene, and (iii) the occlusion or obstruction of the object.

#### 4.6.1. Ambiguity Caused by the Background

The background in the image can sometimes have similar features as the object. In low-light images, the visibility of the edges and textures of the object is degraded, making it more challenging for the tracker to distinguish between the object and the background when they share similar features, hence leading to tracking errors.

**Figure 6:**
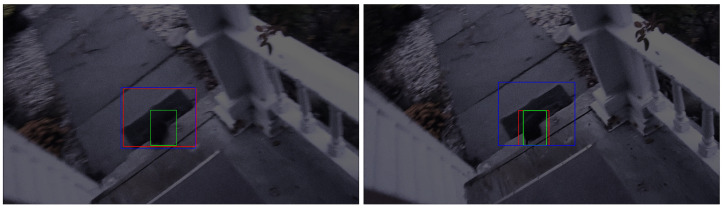
The image on the left shows an example when both trackers fail to track a black cat in the dark due to confusion caused by the background. The image on the right shows an improved result. The green, blue, and red boxes are the ground truth and the results from the models trained with daylight and dark datasets, respectively.

Figure 6 displays two tracking failures, where the tracker struggles to differentiate the black squirrel from the background. The left shows when both the normal and dark trackers fail to identify the object in the frame. The cause of this error is that the door mat is mistaken as the squirrel, as they both appear black and have a slender shape. However, as presented in the image on the right, after the squirrel moves to the doorstep, where its features contract with the background, more features are captured by the dark tracker; hence, it is able to correct the tracking result. On the contrary, the normal tracker continues to fail in recognizing the object in this frame, indicating that its ability in feature extraction in low-light conditions is weaker than that of the dark tracker.

#### 4.6.2. Multiple Objects

The challenges include consistently recognizing individual objects when multiple objects are present in a scene, managing interactions between objects, and coping with the appearance changes of each object.

**Figure 7:**
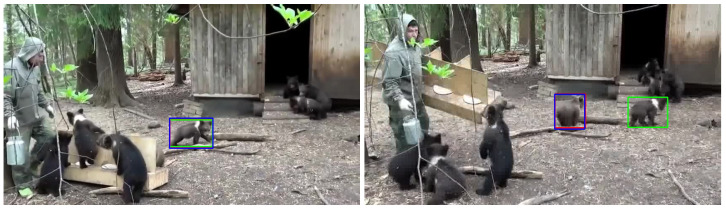
Examples when the normal tracker or both trackers failed to track an object in the dark due to multiple objects existing in the scene. The green, blue, and red boxes are the ground truth and the results from the models trained with daylight and dark datasets, respectively. The images are shown in normal light for better visualization.

Figure 7 presents an example where the trackers are unsuccessful in maintaining the identity of the object. In the two frames, both trackers trained with normal light and low light manage to identify the object (as shown on the left)—a small black bear—when there is only one such bear walking on the ground. Nevertheless, in the right image, both trackers incorrectly identify the original black bear (bear 1) on the right and instead misinterpret the one on the left (bear 2) as the initial bear. This may occur due to the trackers’ inability to accurately follow the object’s movements. Specifically, as bear 1 moves to the right, bear 2 takes its original position. Consequently, despite the trackers’ initial success in tracking the object and the minor changes in the visual appearance of bear 1, they still confuse bear 2 with the originally tracked bear 1. This highlights the trackers’ lack of ability in accurately capturing the temporal information within the sequence. This limitation seems reasonable, considering that the score prediction head in the original model design, which is crucial for capturing temporal information, was trained in the online stage of MixFormer. However, to reduce training complexity in this project, the online training step was excluded when training these trackers. As a result, the trackers may exhibit a diminished ability to capture temporal information.

**Figure 8:**
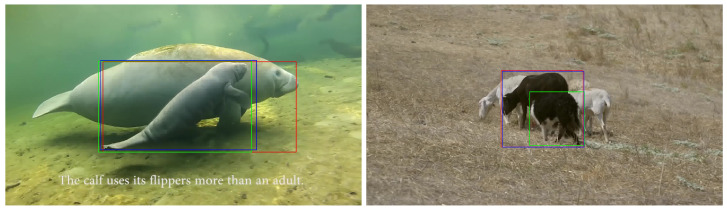
Examples when one or both of the trackers fail to track an object in a frame due to the interaction of objects. The green, blue, and red boxes are the ground truth and the results from the models trained with daylight and dark datasets, respectively. The images are shown in normal light for better visualization.

Figure 8 displays further examples where the trackers fail to track an object due to the presence of multiple objects in the scene. In both cases presented in the frames, the target object is interacting with another object in the scene. Consequently, some features of the other object involved in the interaction are incorrectly attributed to the target object. For example, in the left image, as the calf manatee interacts with the adult manatee, the dark tracker falsely includes the adult manatee’s head as part of the calf. This is probably because the head of the adult manatee has more distinct features, such as the eyes and head shape, which the dark tracker can easily capture. Similarly, in the right image, the border collie is interacting with the black sheep. Since the border collie is in a position where its head is not visible in the picture, the sheep’s head and neck, which have more distinguishable and pronounced features, are mistakenly identified by the trackers as part of the border collie. This mistake can also be attributed to the missing edges of the border collie’s head, making it difficult for the tracker to identify the boundary of the object.

#### 4.6.3. Occlusion

**Figure 9:**
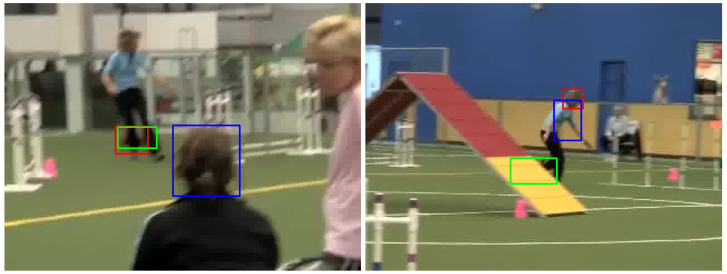
(**Left**): When the object, a black dog, is partially occluded, the dark tracker is able to identify the part that is visible, while the normal tracker cannot identify the object. (**Right**): When the object is fully obstructed, both trackers are unable to track the object in the frame. The green, blue, and red boxes are the ground truth and the results from the models trained with daylight and dark datasets, respectively.

Occlusion poses a considerable challenge in object tracking, as it can impede the tracker’s capacity to maintain a precise representation of the object throughout a sequence [47]. An illustration of this issue can be found in Figure 9, where the trackers struggle to track an object, a black dog, during occlusion events. On the left, the object, a dog, is partially obscured by a person’s legs. In this frame, the dark tracker is able to capture the object’s features and define its edges despite the occlusion. In contrast, the daylight tracker inappropriately identifies the person’s head—which shares similar features, such as a brown and fuzzy appearance, with the dog in the image—as the target. This observation aligns with the previous finding that the dark tracker is more capable at capturing features and defining edges in low-light conditions than the normal tracker, allowing it to identify the object even when occlusion occurs in the dark.

The dark tracker’s ability to handle occlusion has its limitations. In the right frame, where the dog is entirely obscured by the structure, both trackers fail to recognize the animal. This failure can potentially be attributed to the model’s inability to handle temporal information effectively due to the absence of online training, as previously discussed. When a model is adept at processing temporal information, it can leverage the object’s motion patterns, trajectory, and appearance changes observed in previous frames to make predictions about the object’s position and appearance during occlusion [48]. Hence, when the model lacks this ability, it may fail to continuously track the object when the object is partially or entirely hidden from view.

### 4.7. Performance Comparison

This section compares our MixFormer-based tracker with Siam R-CNN [4], VideoTrack [6], and DETR [7]. For a fair comparison, we also integrated the denoiser and enhancement module into all methods. Siam R-CNN merges Siamese networks with region-based convolutional neural networks (R-CNNs). Siam R-CNN excels in robust and accurate visual tracking by effectively matching the target object across frames using a Siamese architecture. The R-CNN component aids in precise object localization and classification. This fusion enhances the tracking performance, especially in challenging scenarios involving occlusions, deformations, and appearance changes. VideoTrack is a newer method, reporting higher accuracy in tracking but requiring greater complexity and memory than MixFormer and Siam R-CNN. VideoTrack extracts spatiotemporal features via a Video Transformer using a hierarchical structure of spatial and temporal attentions. The following segment illustrates the performance comparison between these three methods.

Table 2 displays the outcomes from testing sets generated using various parameters (e.g., sigma, gamma, saturation gain), while other settings remained at their default values (see Section 4.2 for an in-depth explanation of parameter configurations). A noticeable observation in Table 2 is the consistent lower performance of the Siam R-CNN model compared to the MixFormer model in both daylight and low-light tracking scenarios. This observation emphasizes the effectiveness of the MixFormer’s MAM architecture, significantly enhancing tracking performance even under challenging lighting conditions. These results underscore the superiority of the transformer-based architecture over the conventional CNN network and underscore the advantages of the Mixed Attention Module. VideoTrack outperforming MixFormer indicates that hierarchical extraction of spatiotemporal features benefits the tracking performance. However, VideoTrack requires training and testing times approximately three times longer than MixFormer. The results of the three different trackers show that their performances are significantly dropped when performing with dark scenes.

## 5. Conclusions

This study examined the performances of object tracking algorithms in low-light conditions. The strategies involve training the model using synthetic datasets and applying denoising and image enhancement techniques during preprocessing. Our findings demonstrate that training the model on synthetic dark datasets notably improves its performance in low-light settings, particularly under varying noise and brightness levels. Our comprehensive study on the effects of low-light distortions reveals that noise has the most detrimental impact on the tracking performance, followed by non-linear brightness changes. Training the model with a noise level of 25 and a gamma of 0.3 yields the best overall performance across various low-light conditions. Additionally, we proposed and evaluated two preprocessing methods, SUNet for denoising and EnlightenGAN for image enhancement, to enhance the tracking accuracy. Implementing both techniques on the test set results in a 4.41% improvement (AUC) in the tracking accuracy compared to the performance on the noisy dark dataset. Furthermore, utilizing denoising for both the training and testing stages on the dark dataset lead to a 5.36% improvement in the tracking accuracy compared to models trained and tested on the original dark dataset.

## Figures and Tables

**Table 1:** Ablation studies for individual modules within the proposed architecture.

Framework			AUC	OP50	OP75	Precision	Norm Precision
Denoise	Enhance	Tracker					
✗	✗	✓	61.29	73.43	57.60	51.23	72.84
✓	✗	✓	66.65	76.33	59.98	53.01	74.43
✗	✓	✓	64.32	74.93	58.56	52.25	73.21
✓	✓	✓	67.15	77.12	60.72	53.68	75.18

**Table 2:** Test results on different test sets. Dark means a combination of sigma = 40, gamma = 0.5, saturation = 0.4.

Metric	Method	Normal	Sigma = 10	Sigma = 40	Gamma = 0.3	Saturation = 0.3	Dark
AUC	MixFormer	79.30	76.31	73.36	73.17	76.18	67.15
	Siam R-CNN	77.21	74.92	70.24	71.58	74.86	61.93
	VideoTrack	81.16	78.08	75.21	74.74	78.26	68.82
	DETR	78.73	75.23	71.39	71.98	75.57	63.62
Norm Precision	MixFormer	88.93	85.54	83.45	82.35	85.79	75.18
	Siam R-CNN	86.83	84.02	81.73	80.57	85.13	73.65
	VideoTrack	89.74	86.55	84.35	83.16	86.08	77.24
	DETR	87.68	84.07	82.54	81.09	85.15	73.96

## Data Availability

The datasets generated and analyses during the current study are available from the corresponding author upon reasonable request.

## Abbreviations

The following abbreviations are used in this manuscript:

RNN	Recurrent neural network;
CNN	Convolutional neural network;
FCN	Fully convolutional network;
MAM	Mixed attention module;
SPM	Score prediction module;
IoU	Intersection over union;
AUC	Area under the curve.
